# Supplementary material for: Drivers of recent decline in dust activity over East Asia
Source: Nat Commun. 2022 Nov 19;13:7105. doi: 10.1038/s41467-022-34823-3 (PMC9675820; doi:10.1038/s41467-022-34823-3)
Supplement: Supplementary file 1 — Supplementary Information [file 41467_2022_34823_MOESM1_ESM.pdf]

Supplementary information for

Drivers of recent decline in dust activity over East Asia

Chenglai Wu[1*], Zhaohui Lin[1*], Yaping Shao[2], Xiaohong Liu[3], Ying Li[4]

[1]International Center for Climate and Environment Sciences, Institute of Atmospheric Physics, Chinese Academy of Sciences, Beijing 100029, China.

[2]Institute for Geophysics and Meteorology, University of Cologne, Cologne 50969, Germany.

[3]Department of Atmospheric Sciences, Texas A&M University, College Station, TX 77845, USA.

[4]National Climate Center, China Meteorological Administration, Beijing 100081, China.

*Correspondence to: C.W. (wuchenglai@mail.iap.ac.cn); Z.L. (lzh@mail.iap.ac.cn)

This file includes:

Supplementary Methods

Supplementary Notes

Supplementary Tables 1-4

Supplementary Figures 1-23

Supplementary References

Supplementary Methods

Description of dust emission model

The dust emission model (DuEM v1) generally follows the framework of Shao[1] (hereafter Shao04): (1) vertical dust flux is calculated from horizontal (streamwise) sand flux; (2) the horizontal sand flux is a function of friction velocity (u_*) and threshold friction velocity (u_{*t}); (3) u_{*t} is a function of dust particle diameter and depends on soil moisture and surface roughness elements (mainly vegetation). The magnitudes and variations of dust emission fluxes calculated by Shao04 have been rigorously tested against field observations, and the comparison showed a good performance of the scheme[2]. The scheme has also been adopted in regional dust modeling and widely used for the studies of regional dust cycle, dust storm, and air pollution in the regions including North Africa, East Asia, Middle East, and Australia[3-8]. The flow chart of dust emission model is shown in Supplementary Fig. 1.

Shao04 scheme is developed based on wind erosion physics and explicitly calculates dust emission induced by the saltation bombardment (also known as sand blasting) and consequent aggregate disintegration. Suppose that soil particles are divided into I particle size intervals, each with a mean diameter d_i (m) and an increment Δd_i. Then the emission flux for dust in the ith group generated by the saltation bombardment of particles of size d_s (m), $F(d_i, d_s)$ (kg m^{-2} s^{-1}), can be expressed as:

$$F(d_i, d_s) = c_y \eta_{fi} \left((1-\gamma) + \gamma \frac{p_m(d_i)}{p_f(d_i)} \right) \frac{Q(d_s) g}{u_*^2} (1+\sigma_m) \qquad (1)$$

$$\sigma_m = 12 u_*^2 \frac{\rho_b}{p} \left(1 + 14 u_* \sqrt{\frac{\rho_b}{p}}\right) \qquad (2)$$

and the total emission flux for dust in the ith group by all saltation particles (with size from d_1 to d_2) is:

$$\hat{F}(d_i) = \int_{d_1}^{d_2} F(d_i, d_s)\, p(d_s) \delta d_s \qquad (3)$$

In general, particles with diameters smaller than 20 μm can remain suspended for a long period of time in the atmosphere after emission and thus be transported hundreds to thousands of kilometers to affect downwind regions[9]. Therefore, here we calculate the emission of dust particles smaller than 20 μm and use total dust emission flux for analysis.

In Supplementary Equation (1), c_y is a dimensionless coefficient; η_{fi} is the fraction of dust in the ith size group that can be emitted. $p_m(d)$ and $p_f(d)$ (m^{-1}) are the minimally (i.e., raw) and fully disturbed particle size distribution (PSD), respectively. γ describes how easily soil aggregates can be broken and is specified as a function of friction velocity u_* (m s^{-1}) and threshold friction velocity u_{*t} (m s^{-1}):

$$\gamma = \exp\left[-(u_* - u_{*t})^3\right] \qquad (4)$$

The real PSD during dust emission, i.e., $p(d)$ is then calculated as:

$$p(d) = \gamma p_m(d_i) + (1-\gamma) p_f(d_i) \qquad (5)$$

$Q(d_s)$ (kg m^{-1} s^{-1}) is the saltation flux of particles of size d_s; g is gravity acceleration (equal to 9.8 g m^{-2}); σ_m is bombardment efficiency; ρ_b is soil bulk density (equal to 1000 kg m^{-3}); and p is soil plastic pressure (Pa). c_y and p depend on the soil properties[1] and are prescribed for different soil texture types[5,8].

$Q(d_s)$ is calculated using the formulation from White[10], expressed as:

$$Q(d_s) = c_0 \frac{\rho_a}{g} u_*^3 \left(1 - \frac{u_{*t}(d_s)}{u_*}\right)\left(1 + \left(\frac{u_{*t}(d_s)}{u_*}\right)^2\right) \ \text{when } u_* > u_{*t}(d_s) \qquad (6)$$

where c_0 is a coefficient that is set to 2.6 originally[10] but more generally in the order of 1.0[11], and thus c_0 is set to 1.0 here; ρ_a is air density (kg m^{-3}) and is calculated by the model; $u_{*t}(d_s)$ is the threshold friction velocity for particles of size d_s. Note that u_{*t} (Supplementary Equation (4)) is derived from the minima of $u_{*t}(d_s)$ across all diameters d_s. $u_{*t}(d_s)$ is calculated by

$$u_{*t}(d_s) = u_{*t0}(d_s) \times f(\lambda) \times f(w) \qquad (7)$$

$$u_{*t0}(d_s) = \sqrt{a_1\left(\frac{\rho_p}{\rho_a} g d_s + \frac{b_1}{\rho_a d_s}\right)} \qquad (8)$$

In Supplementary Equation (7), $u_{*t0}(d_s)$ is the threshold friction velocity for soil particles of size d_s in the idealized situation when soil is dry and bare. $u_{*t0}(d_s)$ is calculated from Supplementary Equation (8) where a_1 is set to 0.0123 and b_1 is set to 0.0003 kg s^{-1} according to Shao and Lu[12]. λ and w are the frontal area indices of surface roughness elements (mainly vegetation) and volumetric soil moisture in the topsoil layer (m^3 m^{-3}), respectively; and $f(\lambda)$ and $f(w)$ are the corresponding correction functions. $f(\lambda)$ follows Raupach et al.[13]:

$$f(\lambda) = \sqrt{1 - \sigma m \lambda}\sqrt{1 + \beta m \lambda} \qquad (9)$$

where m accounts for the nonuniformity of the surface stress, σ is the ratio of basal-to-frontal-area of the elements, and β is the ratio of the drag coefficient for a single roughness element to that of a surface without roughness elements. Raupach et al.[13] suggested that $\sigma \approx 1$, $\beta \approx 90$, and $m \approx 0.5$ are typical values. λ is a key parameter to describe the geometric feature of surface roughness elements (mainly vegetation). Here λ is derived empirically from leaf area index (LAI) following Shao[9]:

$$\lambda = \gamma \mathrm{LAI} \qquad (10)$$

where γ is a coefficient depending on the characteristics of vegetation and the typical value of 1.0 is used here.

f(w) follows Fecan et al.[14]:

$$f(w) = \sqrt{1 + a_{soil}(w - w_r)^{b_{soil}}} \qquad \text{when } w>w_r \qquad (11)$$

where a_{soil} and b_{soil} are coefficients that depend on the soil texture, and w_r is the threshold volumetric soil moisture (m^3 m^{-3}). Originally Fecan et al.[14] fitted the expression to the experimental data for various soils and recommended a_{soil} =27.72 (=$1.21\times100^{0.68}$), b_{soil}=0.68, and w_r is a function of clay content. The expression is further expanded to several soil texture types by varying a_{soil} and b_{soil} in addition to w_r and a set of values for different soils are recommended by Kang et al.[5]. This set of values from Kang et al.[5] are used here.

As a gridcell can be covered with various surface types and snow, dust emission is multiplied by the fraction of erodible surface (i.e., bare soil). Therefore, the total dust emission flux is calculated as:

$$F = (1 - f_c)(1 - f_{snow}) \sum \hat{F}(d_i) \qquad (12)$$

where f_c is vegetation cover fraction and f_{snow} is snow cover fraction. f_c is assumed to be proportional to LAI using the empirical function:

$$f_c = \frac{\mathrm{LAI}}{\mathrm{LAI_c}} \qquad (13)$$

Where LAI_c is the critical LAI. Here LAI_c is set to 0.3 m^2 m^{-2} following Mahowald et al.[15] and Oleson et al.[16].

To simulate the dust emission over East Asia, input data is provided (Supplementary Fig. 1). The input variables u_*, w, f_{snow}, and ρ_a are from MERRA-2 reanalysis[17]. MERRA-2 provides the soil water content in the top 0-5 cm layer. As soil particles most relevant for dust emission is mainly in the top 1-2 cm layer, a correction factor c_w (i.e., $w = c_w w_{\mathrm{MERRA\text{-}2}}$) is applied to correct the soil water content for dust emission. Here c_w is set to 0.5 following Darmenov[18]. Note that as snow cover fraction is underestimated in MERRA-2 compared to observations, the impacts of snow cover on dust emission may be underestimated in the simulations especially in the cold season[19]. As described above, both f_c and λ is estimated from LAI. $p_m(d)$ and $p_f(d)$ are prescribed from the summation of several log-normal distributions for different soil texture types. Here we use Harmonized World Soil Database (HWSD) soil texture data, which is available at http://www.fao.org/soils-portal/data-hub/soil-maps-and-databases/harmonized-world-soil-database-v12/en/. HWSD soil texture data is provided with 13 United States Department of Agriculture (USDA) types at a resolution of 30-arc. For used in dust emission model, the raw 30-arc soil data is converted into fractional coverage of different soil texture types.

Supplementary Notes

Seasonal variations of dust storm days and comparison with previous studies

During 2001-2017, dust storm occurs most frequently in spring and least frequently in late summer and early autumn. Dust storm also occurs at times in winter and late autumn, with slightly more dust storm days in late winter than in early winter and late autumn (Supplementary Fig. 3).

Most previous studies showed that dust activity in Gobi Deserts and Sandy Lands of northern China is much stronger in spring than in other seasons[20-22]. Our results are consistent with them. Previous studies also showed a second peak of dust activity in winter, which is significantly stronger than that in autumn[20-22]. Compared to these studies, the contrast of dust activity between winter and autumn in our study is smaller, which can be ascribed to the difference in the study periods between our study and these previous studies. Our study focuses on the past two decades, while most previous studies extended the study periods to earlier decades (Supplementary Fig. 3a). Based on a continuous dataset during 1954-2017 (see Methods in the main text), dust activity is significantly weaker for all the four seasons during 2001-2017 than in the previous decades (1960s-1970s and 1980s-1990s), with the most significant decline in spring and winter (Supplementary Fig. 3c, d). Therefore, the stronger decline of dust activity in winter than in autumn in past several decades results in a smaller contrast in the dust storm days between these two seasons during 2001-2017.

Seasonal variations of dust emission and relevant driving factors

The seasonal variations of dust emission are determined by the four driving factors (surface wind speed, soil moisture, vegetation cover, and snow cover) that are all considered in the dust emission model (DuEMv1).

Surface wind speed is highest in spring and lowest in summer (Supplementary Fig. 7a). Soil moisture is lowest during middle spring to early summer (i.e., April to June), and highest in August and September due to the accumulation of precipitation (Supplementary Fig. 7b and 8). In the dust source regions, where runoff is small, soil moisture is mainly determined by the balance between precipitation and evaporation (Supplementary Fig. 8). Although evaporation is largest in summer, precipitation is also largest in this season and the excess of precipitation over evaporation leads to the increase of soil moisture from June to August. Leaf area index (LAI) shows a strong seasonal variation with the largest values in July-August and the smallest values during November to March (Supplementary Fig. 7c). Snow cover shows the strongest seasonal variation, with a peak in January and nearly zero from May to September (Supplementary Fig. 7d).

For the seasonal variations of dust emission, it is highest in spring, corresponding to the highest surface wind speed, relatively low soil moisture (especially in April when soil moisture is lowest during the year), and low LAI in this season. In contrast, dust emission is lowest in summer, due to the smallest surface wind speed, the largest LAI,

and relatively high soil moisture in this season. In autumn and winter, dust emission occurs at times. Although surface wind is stronger in winter than in autumn, dust emission is lower in winter due to much more snow cover in winter than in autumn. Of all the seasons, dust emission is the second largest in autumn.

Seasonal variations of simulated dust emission are mostly consistent with the observations of dust storm days. Although the model overestimates the dust emission in autumn and winter, it captures the strongest dust activity in spring and much weaker dust activity in other seasons. This demonstrates the model's ability in reproducing the seasonal variations of dust activity and the rationality of our methodology. Note that the model overestimates the dust emission from October to February, which may be partly due to the underestimation of snow cover in MERRA-2 reanalysis used in the model[19]. The overestimation of dust emission in autumn and winter can also be ascribed to the fact that the model does not consider the impacts of dead leaves on dust emission. A previous study showed that dead leaves can serve as a major type of roughness elements after the growing season and effectively reduce the dust emission in East Asia[23].

Supplementary Tables

Supplementary Table 1. Definition of dust events corresponding to present weather (ww) reported from a manned weather station. Note that ww ranges from 00-99 and only those indicating the occurrence dust events are reported here. The full version of ww can be found in WMO-No. 306[24].

**Dust events**	**Present weather (ww)**	**Definition**	**Visibility**
Dust-in-suspension (DIS)	06	Widespread dust in suspension in the air, not raised by wind at or near the station at the time of observation	Usually not greater than 10 km
Blowing dust (BD)	07	Dust or sand raised by wind at or near the station at the time of observation, but no well-developed dust whirl(s) or sand whirl(s), and no duststorm or sandstorm seen	1-10 km
Dust storm (DS)	09	Duststorm or sandstorm within sight at the time of observation, or at the station during the preceding hour	0.5-1 km
	30	Slight or moderate duststorm or sandstorm has decreased during the preceding hour	
	31	Slight or moderate duststorm or sandstorm with no appreciable change during the preceding hour	
	32	Slight or moderate duststorm or sandstorm has begun or has increased during the preceding hour	
	33	Severe duststorm or sandstorm has decreased during the preceding hour	<0.5 km
	34	Severe duststorm or sandstorm with no appreciable change during the preceding hour	
	35	Severe duststorm or sandstorm has begun or has increased during the preceding hour	

Supplementary Table 2. Dust emission during 2001 and 2010s (2010-2017) and the changes from 2001 to 2010s (2010-2017) over Eastern Sources. The unit is Tg yr^{-1} for annual total (ANN) and Tg $season^{-1}$ for each season (MAM, JJA, SON, and DJF) The four experiments are listed in Table 1.

**Exp. name**	**2001**					**2010-2017**				
	**ANN**	**MAM**	**JJA**	**SON**	**DJF**	**ANN**	**MAM**	**JJA**	**SON**	**DJF**
Baseline (All: Wind+LAI+SOILM)	310	198	34	24	54	202	103	26	39	34
Wind	310	198	34	24	54	254	131	37	46	40
LAI	310	198	34	24	54	273	177	27	20	49
SOILM	310	198	34	24	54	281	178	28	25	50
	**Changes from 2001 to 2010s (2010-2017)**					**Relative contributions to the total changes (%)[a]**				
	**ANN**	**MAM**	**JJA**	**SON**	**DJF**	**ANN**	**MAM**	**JJA**	**SON**	**DJF**
Baseline (All: Wind+LAI+SOILM)	-108	-95	-8	15	-20	100%	100%	100%	100%	100%
Wind	-56	-67	3	22	-14	46%	62%	-30%[b]	116%	61%
LAI	-37	-21	-7	-4	-5	30%	19%	70%	-21%[b]	22%
SOILM	-29	-20	-6	1	-4	24%	19%	60%	5%	17%

[a]: The relative contribution from each factor (Wind, LAI, SOILM) is calculated by dividing the individual change due to the factor to the sum of all the individual changes due to the three factors. For example, 46% is derived by dividing -56 by -122 (=-56+(-37)+(-29)).

[b]: The negative contributions indicate that the change caused by an individual factor is opposite in sign to the total change by all the three factors.

Supplementary Table 3. The 17 land cover types based on International Geosphere-Biosphere Programme (IGBP) classification scheme for MCD12C1 product

**Name**	**Value**	**Description**
Water Bodies	0	At least 60% of area is covered by permanent water bodies.
Evergreen Needleleaf Forests	1	Dominated by evergreen conifer trees (canopy >2m). Tree cover >60%.
Evergreen Broadleaf Forests	2	Dominated by evergreen broadleaf and palmate trees (canopy >2m). Tree cover >60%.
Deciduous Needleleaf Forests	3	Dominated by deciduous needleleaf (larch) trees (canopy >2m). Tree cover >60%.
Deciduous Broadleaf Forests	4	Dominated by deciduous broadleaf trees (canopy >2m). Tree cover >60%.
Mixed Forests	5	Dominated by neither deciduous nor evergreen (40-60% of each) tree type (canopy >2m). Tree cover >60%.
Closed Shrublands	6	Dominated by woody perennials (1-2m height) >60% cover.
Open Shrublands	7	Dominated by woody perennials (1-2m height) 10-60% cover.
Woody Savannas	8	Tree cover 30-60% (canopy >2m).
Savannas	9	Tree cover 10-30% (canopy >2m).
Grasslands	10	Dominated by herbaceous annuals (<2m).
Permanent Wetlands	11	Permanently inundated lands with 30-60% water cover and >10% vegetated cover.
Croplands	12	At least 60% of area is cultivated cropland.
Urban and Built-up Lands	13	At least 30% impervious surface area including building materials, asphalt, and vehicles.
Cropland/Natural Vegetation Mosaics	14	Mosaics of small-scale cultivation 40-60% with natural tree, shrub, or herbaceous vegetation.
Permanent Snow and Ice	15	At least 60% of area is covered by snow and ice for at least 10 months of the year.
Barren	16	At least 60% of area is non-vegetated barren (sand, rock, soil) areas with less than 10% vegetation.

Supplementary Table 4. Comparison in key aspects between this study and the Tai et al.[25] study

**Aspects**	**This study**	**The Tai et al.[25] study**
	Main focus	
Objective	Isolate the impacts of surface wind speed, soil moisture, snow cover, and vegetation cover on the variations of dust emission.	Isolate the impacts of climatic factors (including surface wind, precipitation, temperature) and vegetation cover on the variations of dust emission.
Period	2001-2017	1982-2010
	Methodology	
Sensitive experiments	Impacts of surface wind speed, vegetation cover, soil moisture and snow cover are separated with each factor fixed in 2001.	Impacts of climatic factors are combined together and separated from those of land cover change with meteorology or land cover fixed in 1995. Regression analysis is then used to determine the contributions of each climatic factor
	Modeling framework	
Model	A physically-based dust emission model (DuEM v1)	A chemical transport model (GEOS-Chem) with a physically-based dust emission module[26]
Parameterizations on the impacts of vegetation cover on dust emission	The impacts of vegetation cover change on both bare soil fraction (Supplementary Equations (12)-(13) and threshold friction velocity are considered (Supplementary Equations (9)-(10)	The impacts of vegetation cover change on threshold friction velocity are considered (Equation 3 of Tai et al.[25]) and bare soil fraction is set to be constant.
Meteorological forcing	MERRA-2[17]	MERRA[27]
LAI data*	MODIS[28,29]	Fusion of MODIS (2000-2011) and AVHRR (1982-2010)[30]
Horizontal resolution	$0.5^{o}\times0.625^{o}$	$2^{o}\times2.5^{o}$, with subgrid wind distribution based on MERRA wind at $0.5^{o}\times0.666^{o}$
Model output	Dust emission	Dust emission, dust concentrations, dust deposition, dust optical depth
	Model validation	
Comparison of	Simulated dust emissions are	Simulated dust optical depth is

simulation and observations	compared with the dust storm records at synoptic stations in terms of spatial and temporal variations	compared to satellite observations during 2000-2010 (excluding 2001) and three surface stations.
Main conclusions		
Role of climatic factors and vegetation cover	The weakening of surface wind and the increasing of vegetation cover and soil moisture have all contributed to the decline in dust activity during 2001 to 2017. The relative contributions of these three factors to the dust emission reduction during 2010-2017 relative to 2001 are 46%, 30%, and 24%, respectively.	The interannual variability and trends of East Asian dust emission are largely shaped by climatic factors, with the vegetation cover playing secondary but locally important roles especially in the semiarid and non-desert regions undergoing rapid land cover change and desertification; Of all the climatic factors, surface wind speed, followed by total precipitation, was the most important meteorological factor controlling dust variability and trends, with the weakening of wind playing the largest role in the overall decline.

*: Better quality LAI is obtained by MODIS than by AVHRR[31].

Supplementary Figures

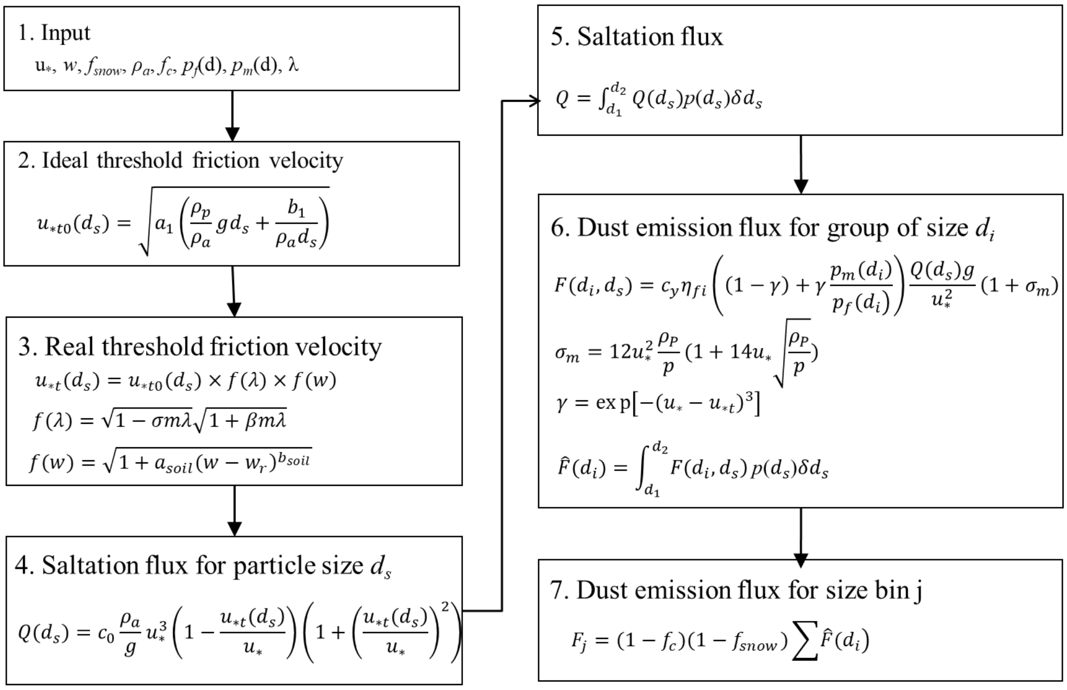

Supplementary Figure 1. The flow chart of dust emission model (DuEM v1) used in this study.

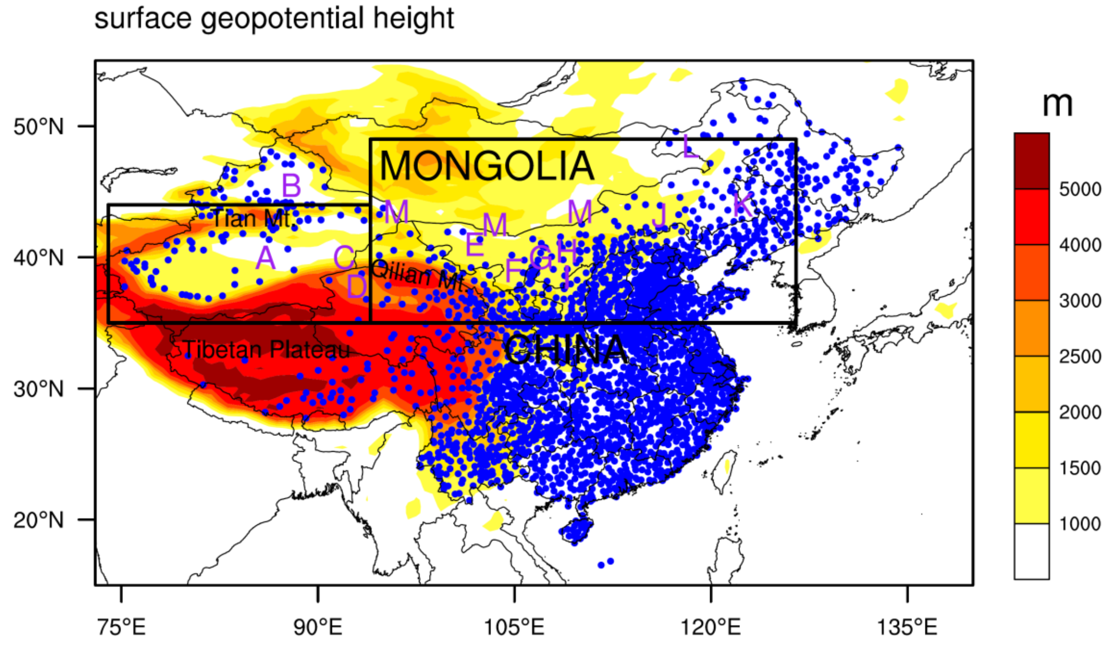

Supplementary Figure 2. Surface synoptic stations in China where dust events are recorded every 3 hours. In total, there are 2340 stations. The color bar denotes the terrain height. The two black rectangles denote the Western Sources consisting mostly of Tarim Basin (35-44 ºN, 74-94 ºE) and the Eastern Sources (35-49 ºN, 94-126.5 ºE), respectively. Purple capitals denote the main deserts in East Asia: A, Taklimakan Desert (over Tarim Basin); B, Gurbantunggut Desert (over Junggar Basin); C, Kumtag Desert; D, Qaidam Basin; E, Badain Juran Desert; F, Tengger Desert; G, Ulan Buh Desert; H, Hobq Desert; I, Mu Us Sandy Land; J, Otindag Sandy Land; K, Horqin Sandy Land; L, Hulun Buir Sandy land; M, Gobi Deserts.

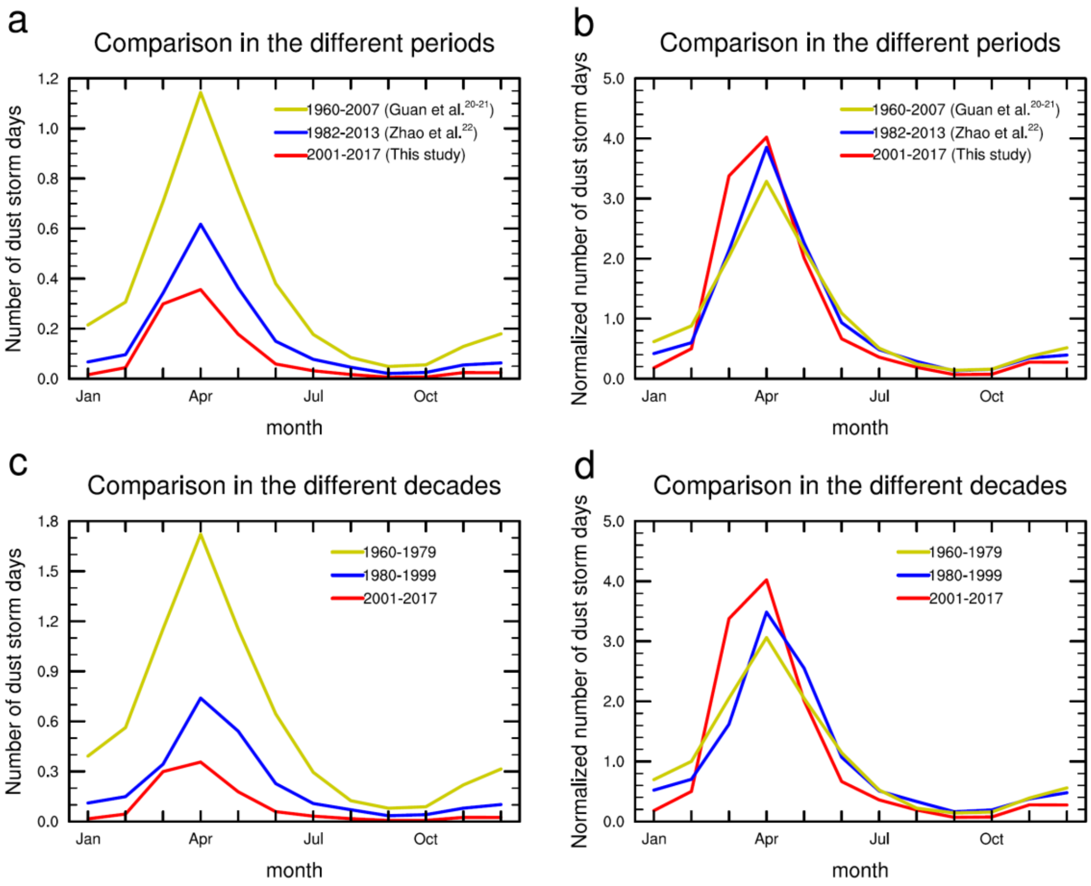

Supplementary Figure 3. Seasonal variations of dust storm days (N_{DS}) and normalized N_{DS} (normalized by the mean of N_{DS} for each curve) in different periods. (**a**) N_{DS} and (**b**) normalized N_{DS} for the three periods examined in Guan et al.[20-21] (1960-2007), Zhao et al.[22] (1982-2013), and this study (2001-2017), respectively. (**c**) N_{DS} and (**d**) normalized N_{DS} for 1960s-1970s (1960-1979), 1980s-1990s (1980-1999), and this study (2001-2017), respectively. All the results are derived from a continuous dataset from 1950s to 2010s (see Methods in the main text).

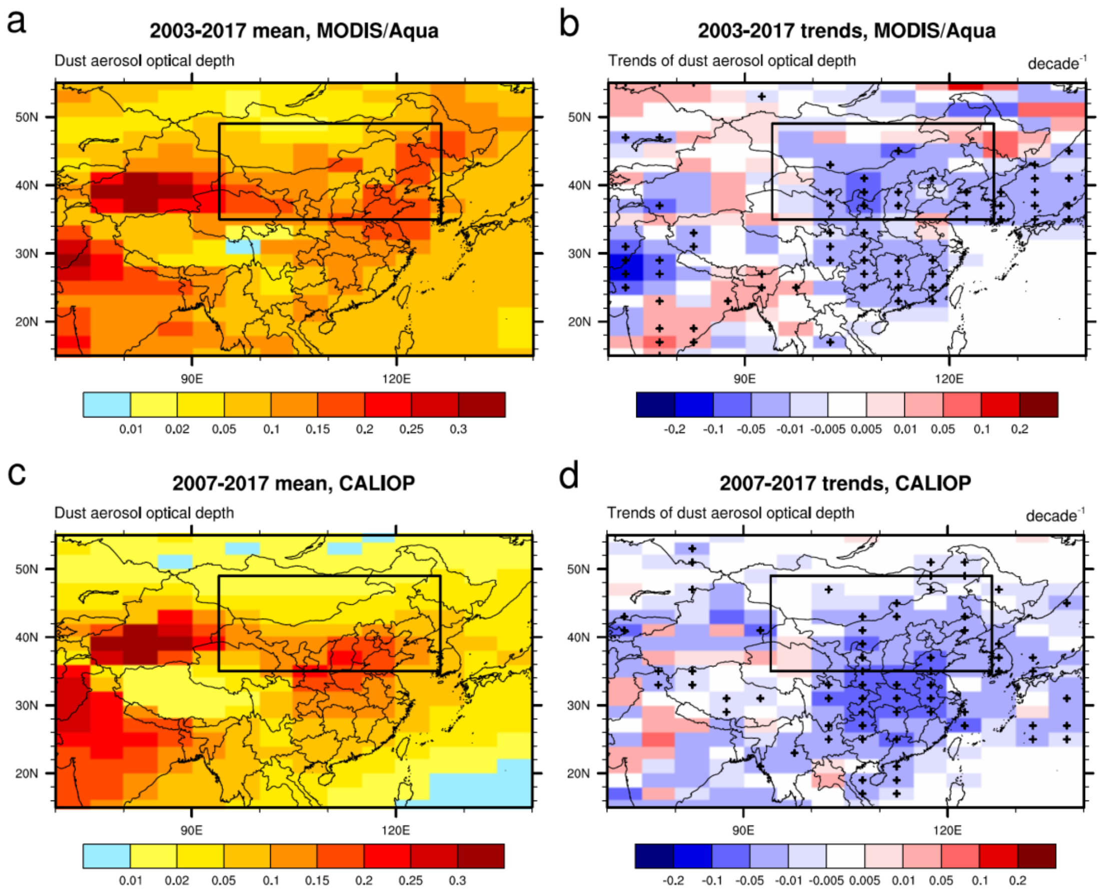

Supplementary Figure 4. Annual mean dust aerosol optical depth (DAOD) and its trends. (**a**) Moderate Resolution Imaging Spectroradiometer aboard the Aqua (MODIS/Aqua) DAOD and (**b**) its trends. (**c**) Cloud-Aerosol Lidar with Orthogonal Polarization (CALIOP) DAOD and (**d**) its trends. Both MODIS/Aqua and CALIOP observations are available at https://drive.google.com/drive/folders/1aQVupe7govPwR6qmsqUbR4fJQsp1DBCX?usp=sharing[32]. The horizontal resolution of both two products are 2° (latitude) × 5° (longitude). The observation period is 2003-2017 for MODIS/Aqua and 2007-2017 for CALIOP. The details of the dust detection algorithms for these two products can be found in Song et al.[32] and references therein. Note that MODIS/Terra is not used as its retrievals may generate the spurious dust trend[32,33]. The crosses denote the regions where the trends are statistically significant (p<0.1). The black rectangle denotes the Eastern Sources (35-49 ºN, 94-126.5 ºE).

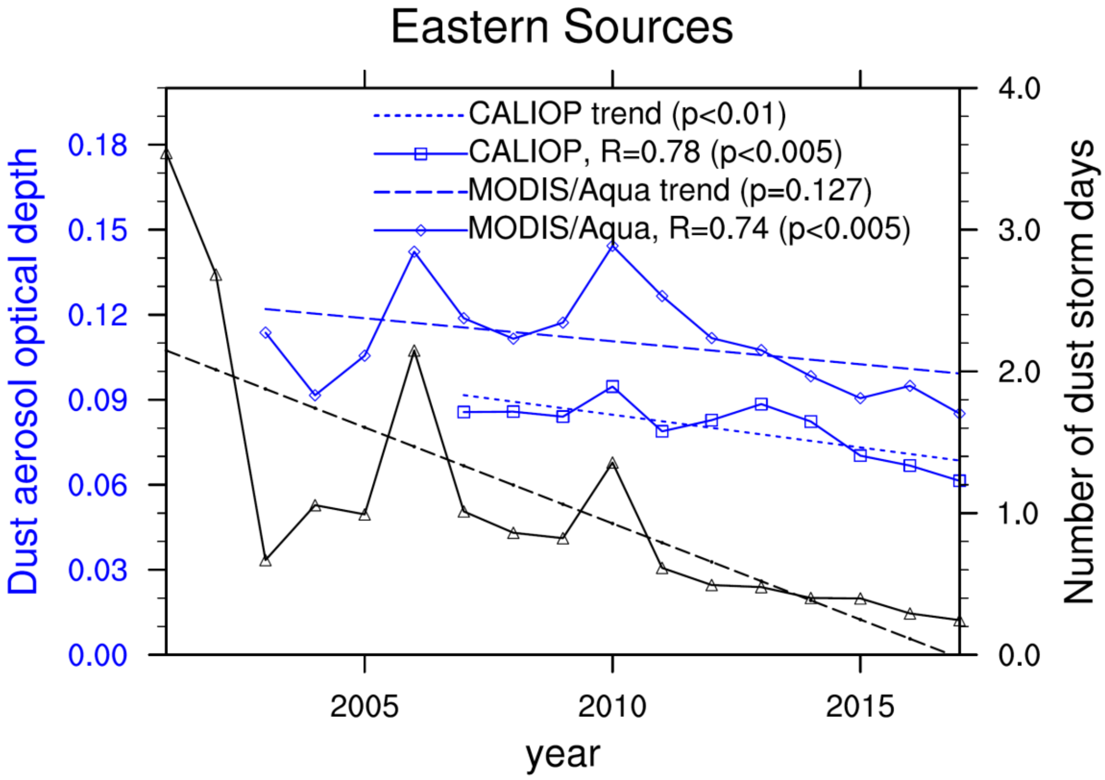

Supplementary Figure 5. The temporal variations of regional mean dust aerosol optical depth (DAOD; left axis; blue) and dust storm days (N_{DS}; right axis; black) during 2001-2017 in Eastern Sources. The correlation between DAOD and N_{DS} is 0.74 (p<0.005) for MODIS/Aqua and 0.78 (p<0.005) for CALIOP. Also shown are the linear trends (dash lines; p<0.001 for N_{DS}, p<0.01 for CALIOP DAOD and p=0.127 for MODIS/Aqua DAOD). Note that MODIS/Aqua and CALIOP observations are available from 2003-2017 and from 2007-2017, respectively. Also note that MODIS/Aqua DAOD trends show a less significant decreasing trend than CALIOP DAOD, which can be explained by the fact that MODIS/Aqua began in the years (2003-2005) when there is a relatively lower DAOD than in the following years (2006-2010) but after 2010s, MODIS/Aqua DAOD significantly decreases.

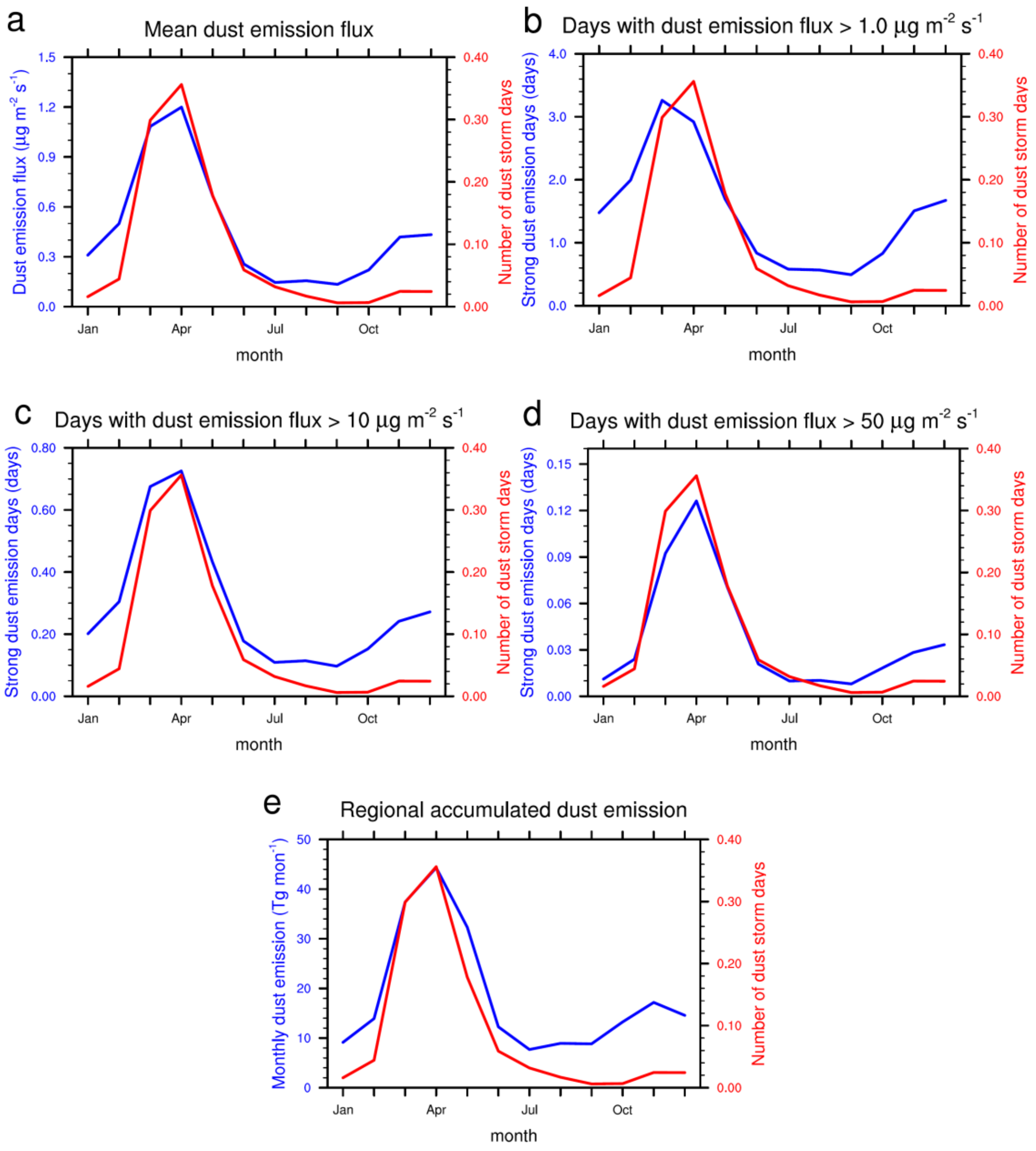

Supplementary Figure 6. Seasonal variations of dust storm days and simulated dust emission flux/days/amount. Seasonal variations of dust storm days (right axis) and simulated dust emission flux (**a**; left axis; µg m^{-2} s^{-1}) and days (**b**, **c**, **d**; left axis) averaged over the synoptic stations during 2001-2017 for Eastern Sources. Also shown are regionally accumulated dust emission (**e**; left axis; Tg mon^{-1}) during 2001-2017 for Eastern Sources. Note the dust emission day is defined as a day with daily mean dust emission flux exceeding 1 µg m^{-2} s^{-1} (**b**), 10 µg m^{-2} s^{-1} (**c**), or 50 µg m^{-2} s^{-1} (**d**). The values of 1 and 10 µg m^{-2} s^{-1} are selected according to the field observations of Shao et al.[2], which showed the low end of observed dust emission flux for particles smaller than 8.4 µm in diameter mostly lies within 1-10 µg m^{-2} s^{-1}. In this study, the model calculates the dust emission flux for particles in a larger size range (i.e., smaller than 20 µm in diameter), and thus in addition to the values of 1 and 10 µg m^{-2} s^{-1}, a larger value (i.e., 50 µg m^{-2} s^{-1}) is also used.

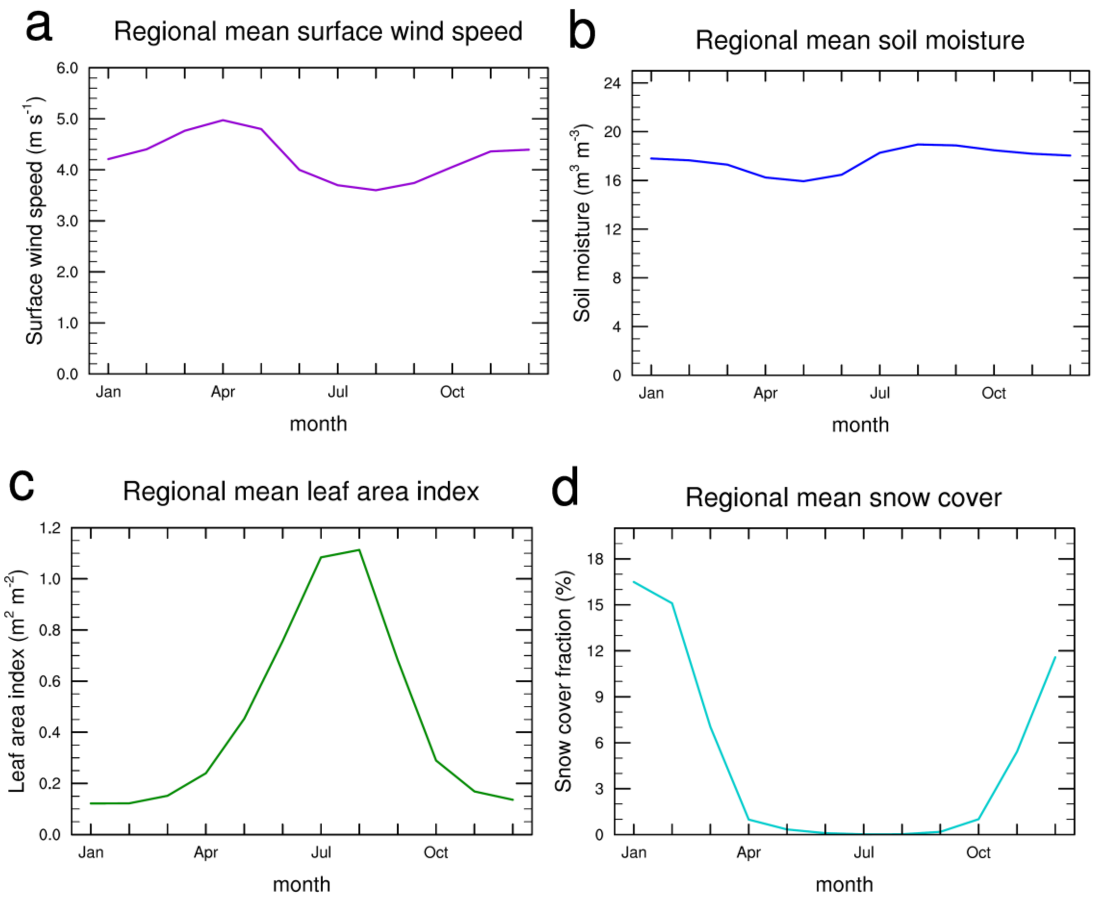

Supplementary Figure 7. Seasonal variations of associated factors for dust emission. (**a**) Surface wind speed. (**b**) Soil moisture in the top 0-5 cm layer. (**c**) Leaf area index. (**d**) Snow cover fraction. The results are all averaged over the Eastern Sources during 2001-2017 from MERRA-2 reanalysis.

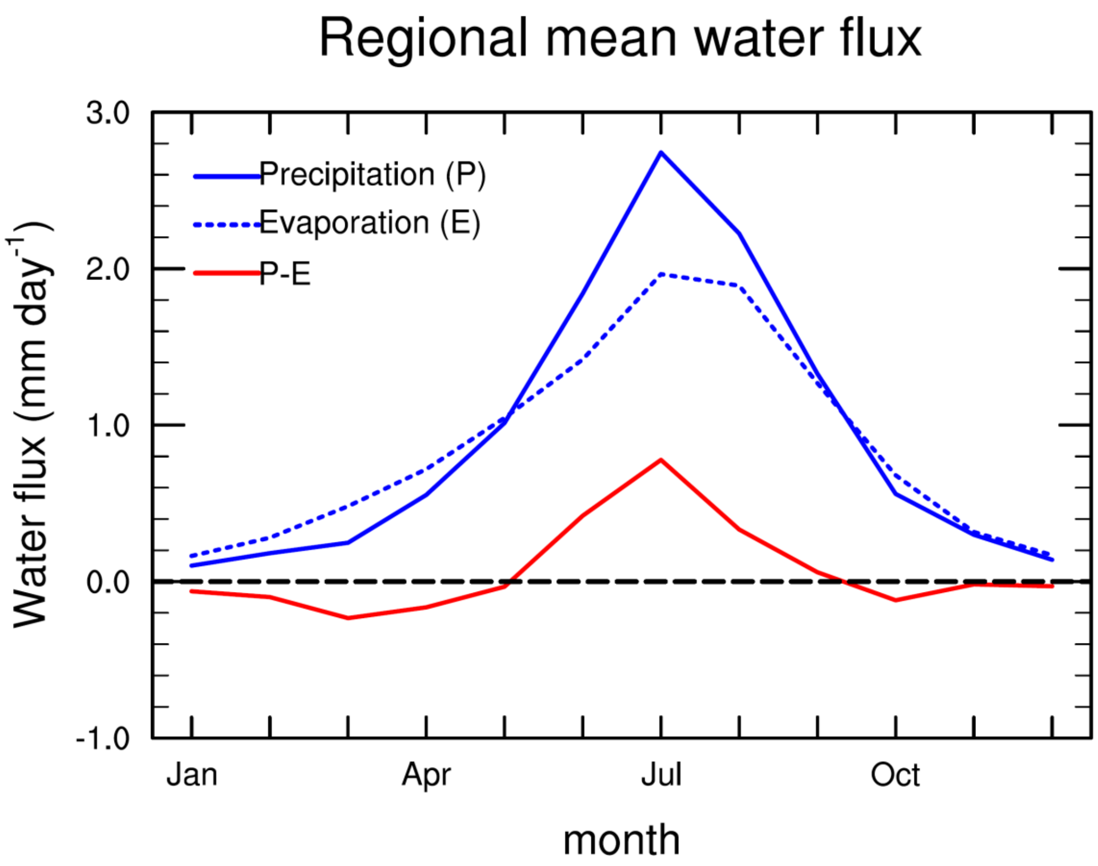

Supplementary Figure 8. Seasonal variations of precipitation (P), evaporation (E), and their difference (P-E) averaged over the Eastern Sources during 2001-2017 from MERRA-2 reanalysis. Note that the precipitation used to force the land surface model has been corrected by observations in MERRA-2[19,34] and is shown here.

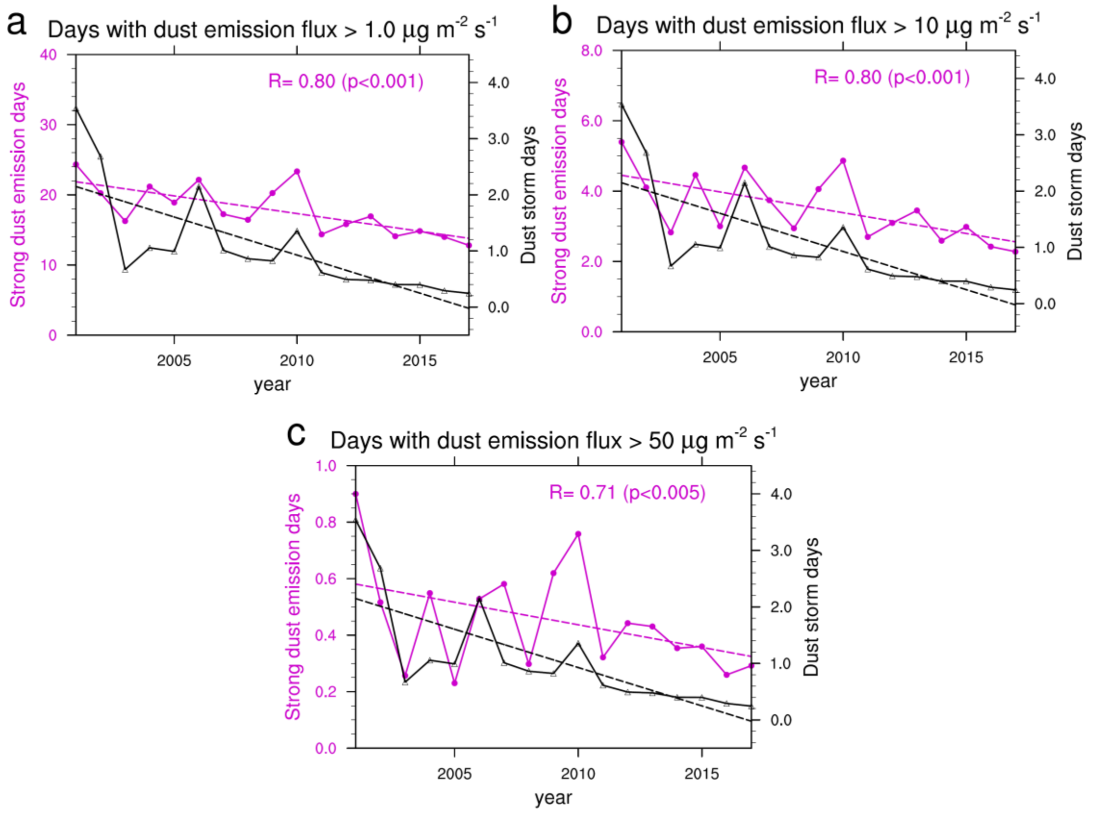

Supplementary Figure 9. The temporal variations of observed dust storm days (right axis) and simulated dust emission days (left axis) averaged over the synoptic stations during 2001-2017 for Eastern Sources. The dust emission day is defined as a day with daily mean dust emission flux exceeding (**a**) 1 μg m^{-2} s^{-1}, (**b**) 10 μg m^{-2} s^{-1}, or (**c**) 50 μg m^{-2} s^{-1}, as described in Figure S6. The correlation between simulation and observations is 0.80 (**a**; p<0.001), 0.80 (**b**; p<0.001), and 0.71 (**c**; p<0.005), respectively.

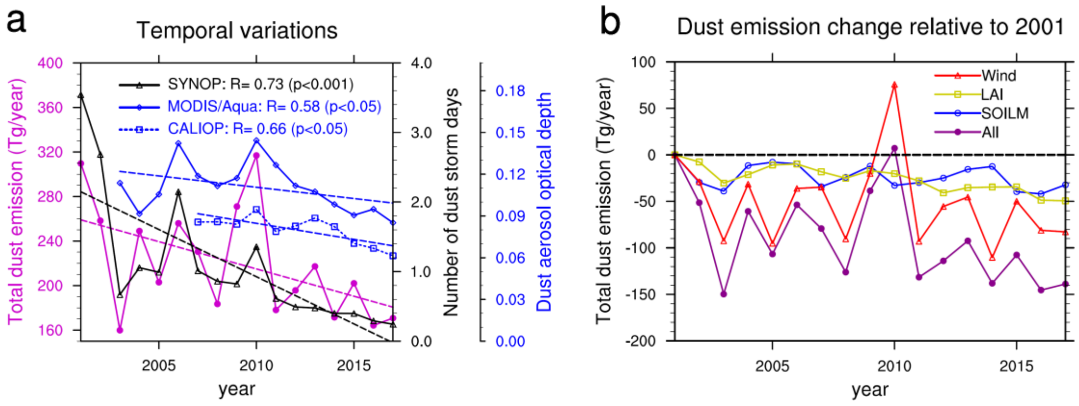

Supplementary Figure 10. Temporal variations of dust storms days, dust aerosol optical depth (DAOD), and regional accumulated emission during 2001-2017 in Eastern Sources. (**a**) Regional mean dust storm days from synoptic observations ("SYNOP"), DAOD from MODIS/Aqua and CALIOP, and regional accumulated dust emission (from baseline experiment; Tg yr^{-1}). The correlation between simulation and SYNOP/MODIS/CALIOP observation is 0.73 (p<0.001), 0.58 (p<0.05), and 0.66 (p<0.05), respectively. Also shown are the linear trends (dash lines; p<0.05 for simulation, p<0.001 for SYNOP, p<0.01 for CALIOP and p=0.127 for MODIS/Aqua). Note that MODIS/Aqua and CALIOP observations are available from 2003-2017 and from 2007-2017, respectively. (**b**) Regional accumulated dust emission (Tg yr^{-1}) during 2001-2017 relative to 2001 from various experiments (Table 1). The standard deviation is 50 Tg yr^{-1} (baseline, "All"), 46 Tg yr^{-1} ("Wind"), 14 Tg yr^{-1} ("LAI"), and 13 Tg yr^{-1} ("SOILM").

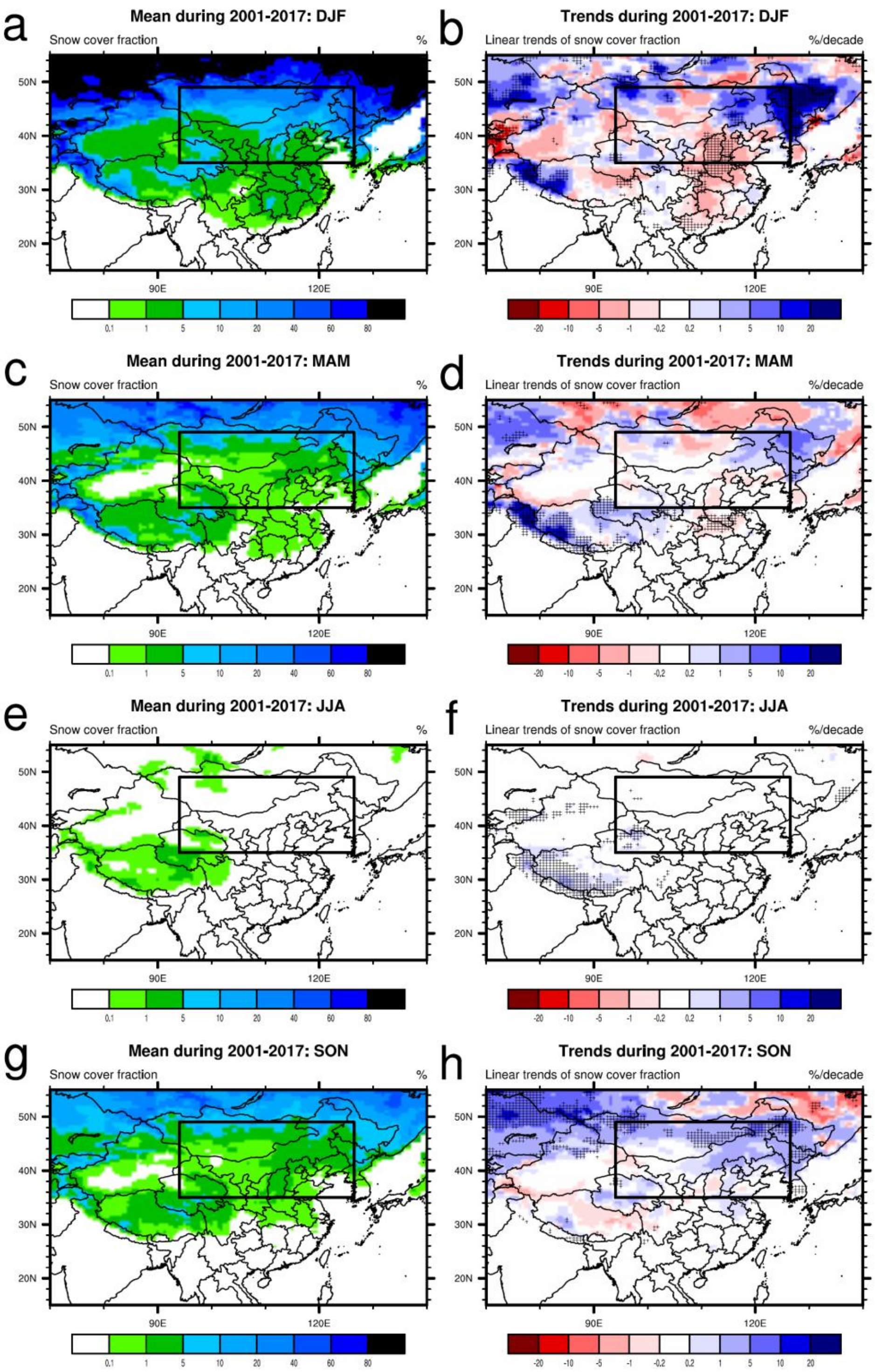

Supplementary Figure 11. Seasonal mean snow cover fractions (%) and its linear trends (% decade^{-1}) during 2001-2017 from MERRA-2 data. Seasonal mean: (**a**) winter (December-January-February: DJF), (**c**) spring (March-April-May, MAM), (**e**) summer (June-July-August, JJA), and (**g**) autumn (September-October-November, SON). Linear trends: (**b**) winter, (**d**) spring, (**f**) summer, and (**h**) autumn. The crosses denote the regions where the trends are statistically significant (p<0.1). The black rectangle denotes the Eastern Sources (35-49 ºN, 94-126.5 ºE).

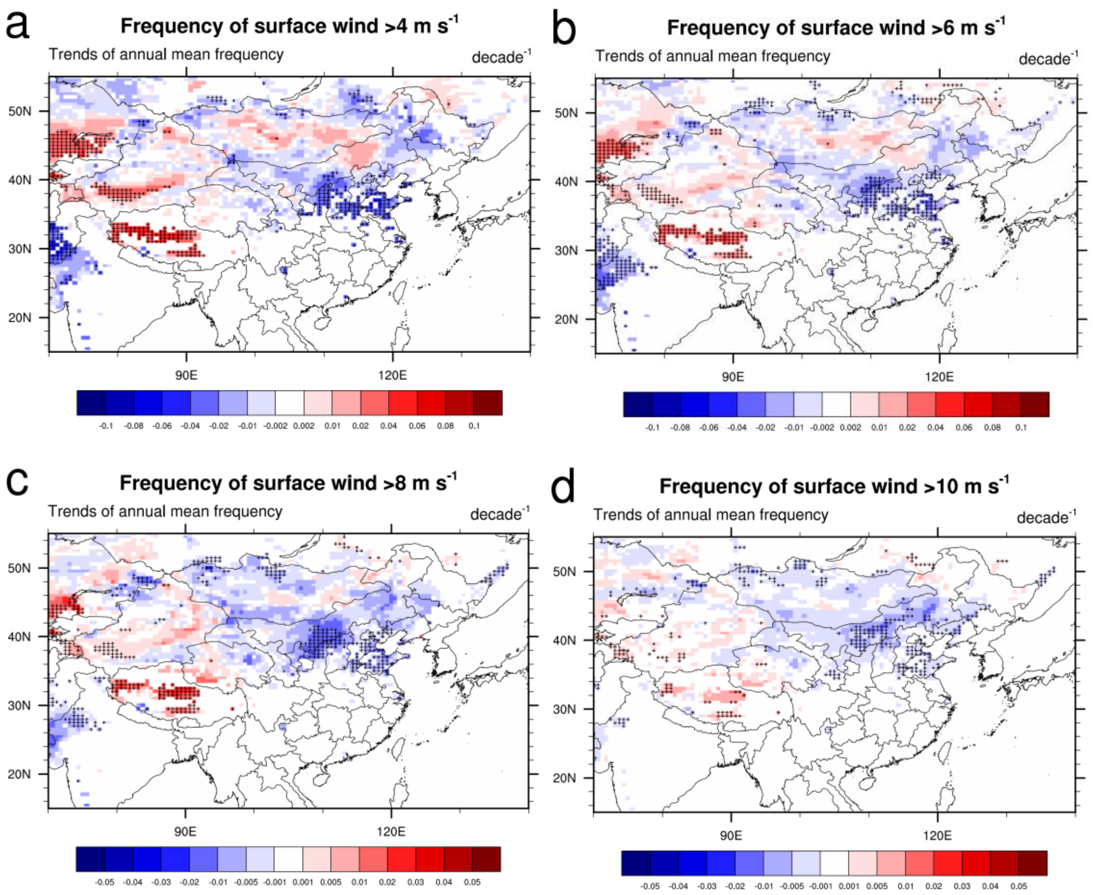

Supplementary Figure 12. Linear trends (decade^{-1}) of frequency of surface wind speed with different magnitudes during 2001-2017. (a) Surface wind speed > 4 m s^{-1}. (b) Surface wind speed > 6 m s^{-1}. (c) Surface wind speed > 8 m s^{-1} (d) Surface wind speed > 10 m s^{-1}. The trends are shown only in the dust emission regions (mean dust emission flux > 0.001 g m^{-2} yr^{-1}). The crosses denote the regions where the trends are statistically significant (p<0.1).

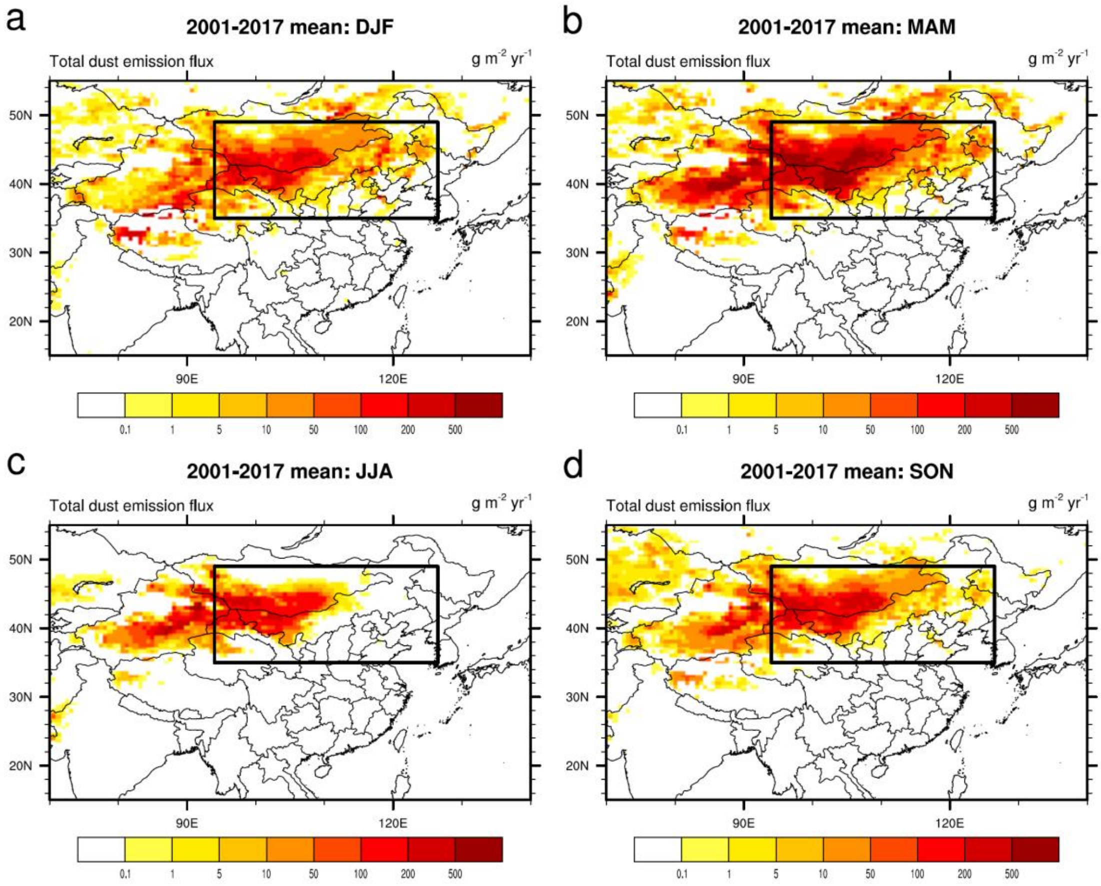

Supplementary Figure 13. Seasonal mean dust emission flux (F_{emis}; g m^{-2} yr^{-1}) during 2001-2017 calculated by the dust emission model (from the baseline experiment). (**a**) Winter (DJF). (**b**) Spring (MAM). (**c**) Summer (JJA). (**d**) Autumn (SON). The black rectangle denotes the Eastern Sources (35-49 ºN, 94-126.5 ºE).

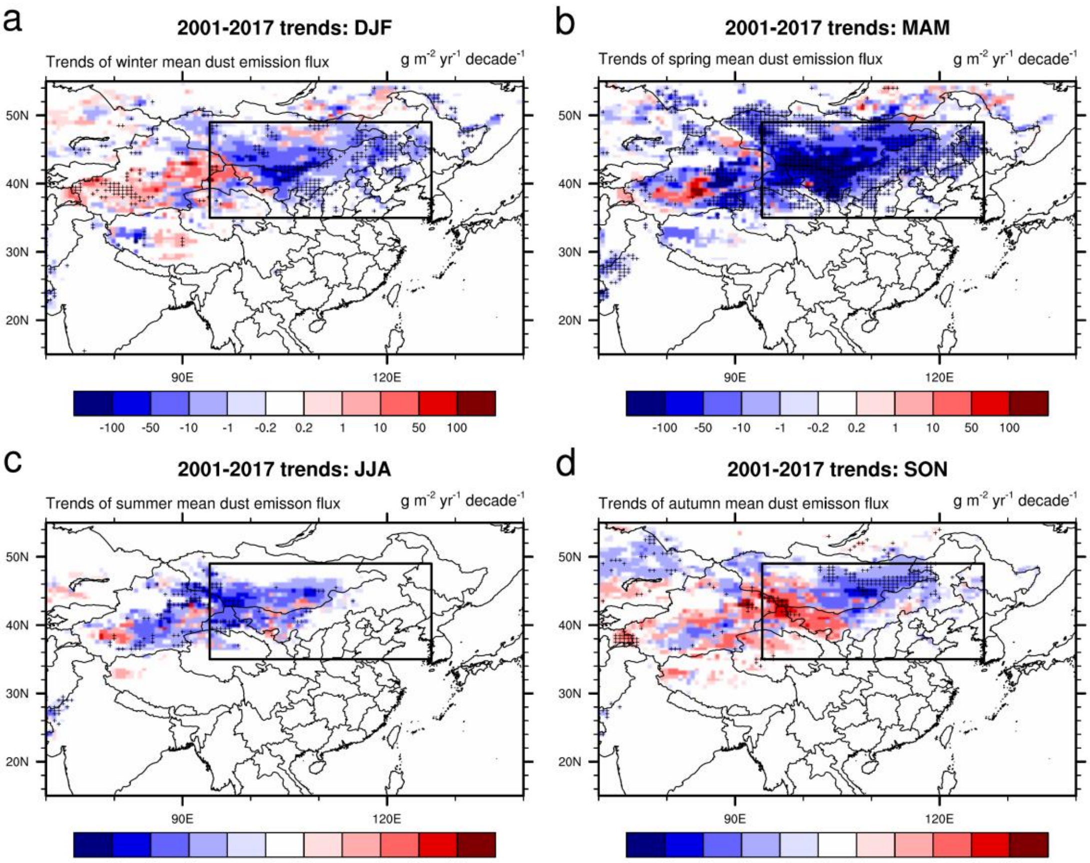

Supplementary Figure 14. Trends of dust emission flux in four seasons based on the baseline experiment (*p*<0.1 for regions marked with crosses). The black rectangle denotes the Eastern Sources (35-49 ºN, 94-126.5 ºE).

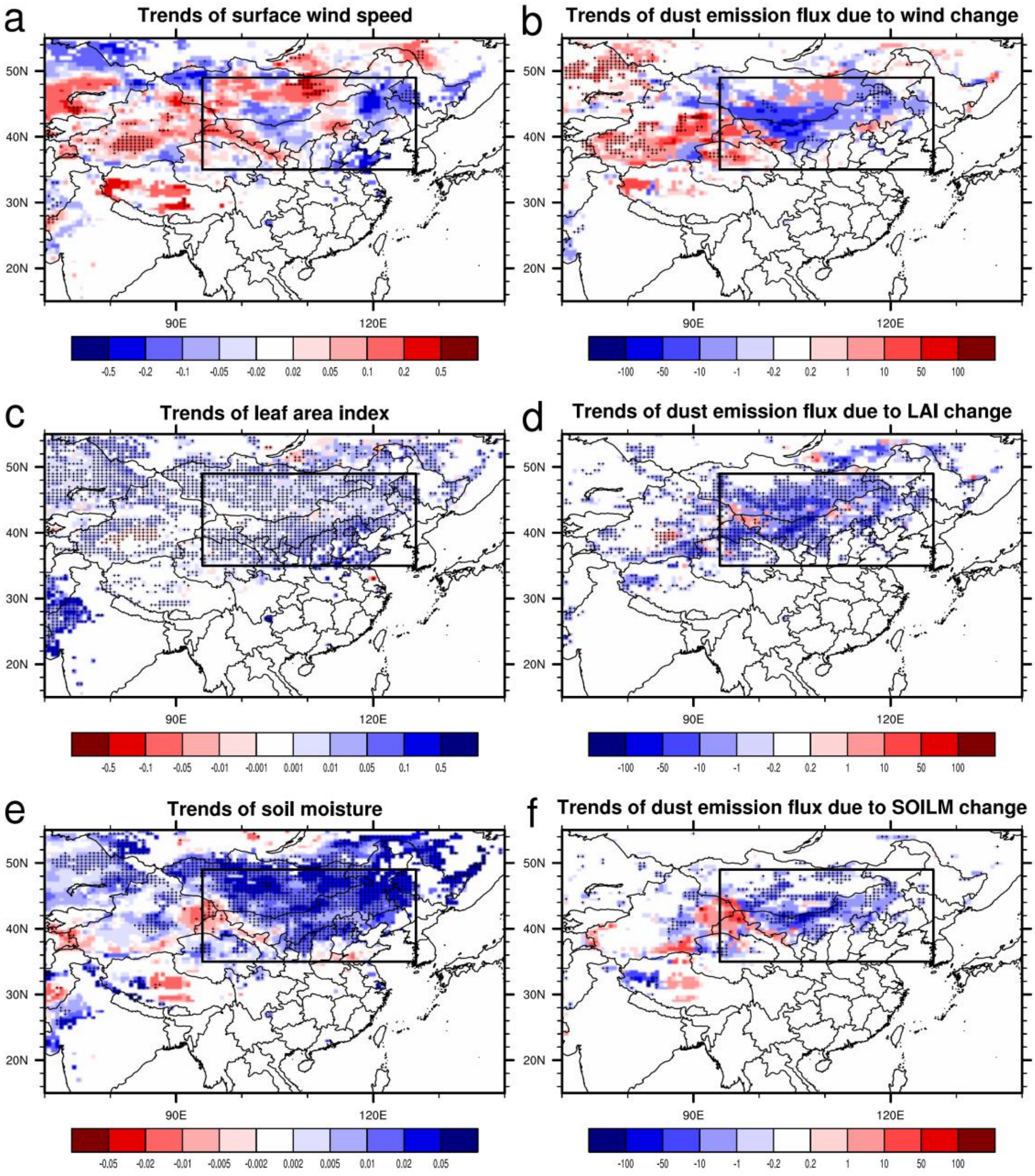

Supplementary Figure 15. Trends of surface wind speed, leaf area index (LAI), soil moisture, and dust emission flux for winter. Linear trends of (**a**) seasonal mean surface wind speed (m s^{-1} $decade^{-1}$), (**c**) leaf area index (m^2 m^{-2} $decade^{-1}$), and (**e**) soil moisture (m^3 m^{-3} $decade^{-1}$) during 2001-2017. The trends are shown only in the dust emission regions (mean dust emission flux > 0.001 g m^{-2} yr^{-1}). Linear trends of dust emission flux (g m^{-2} yr^{-1} $decade^{-1}$) during 2001-2017 due to (**b**) changes of surface wind speed (Wind), (**d**) leaf area index (LAI), and (**f**) soil moisture (SOILM), respectively. The crosses denote the regions where the trends are statistically significant (p<0.1). The black rectangle denotes the Eastern Sources (35-49 ºN, 94-126.5 ºE).

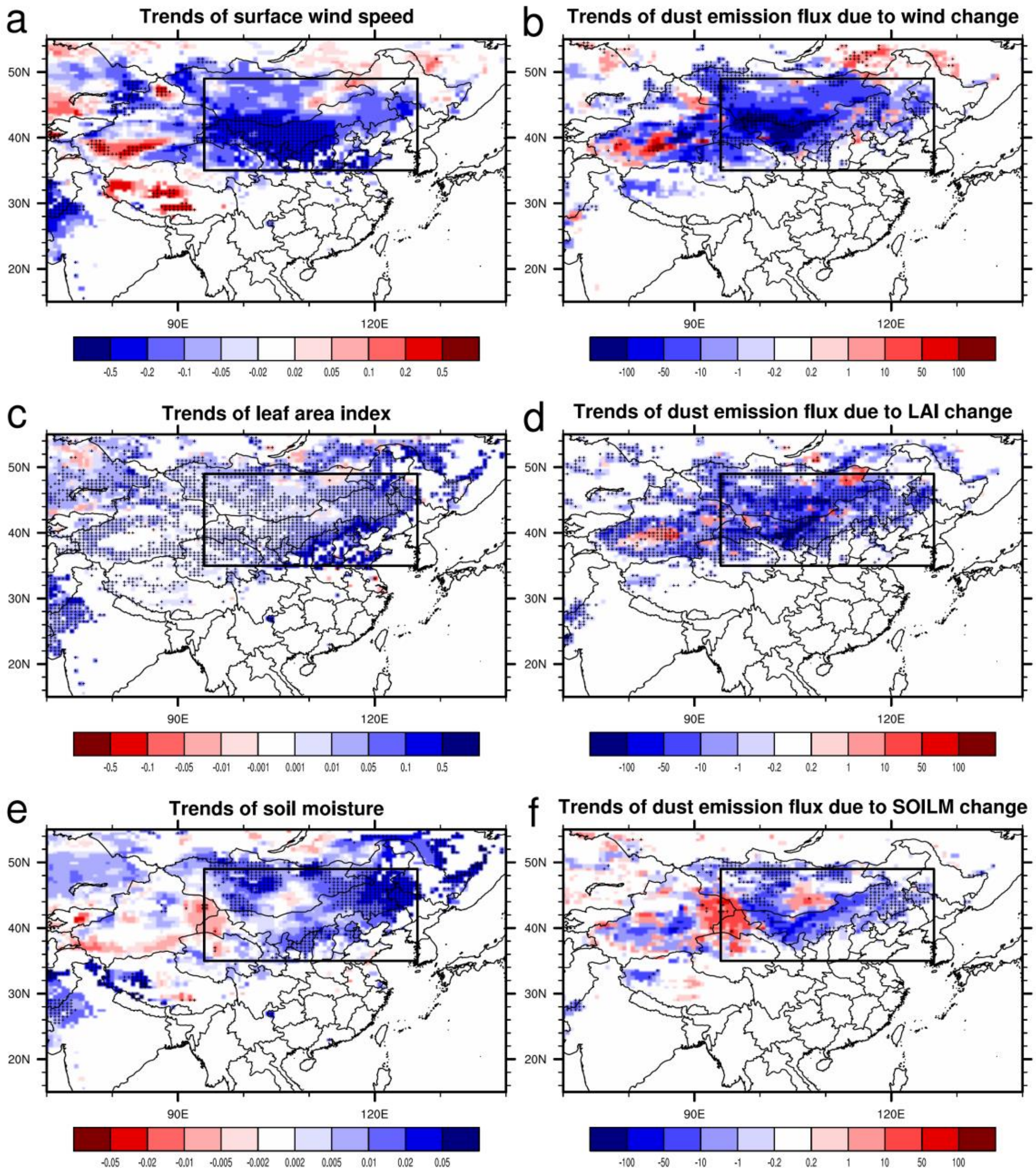

Supplementary Figure 16. Same as Figure S15, but for spring.

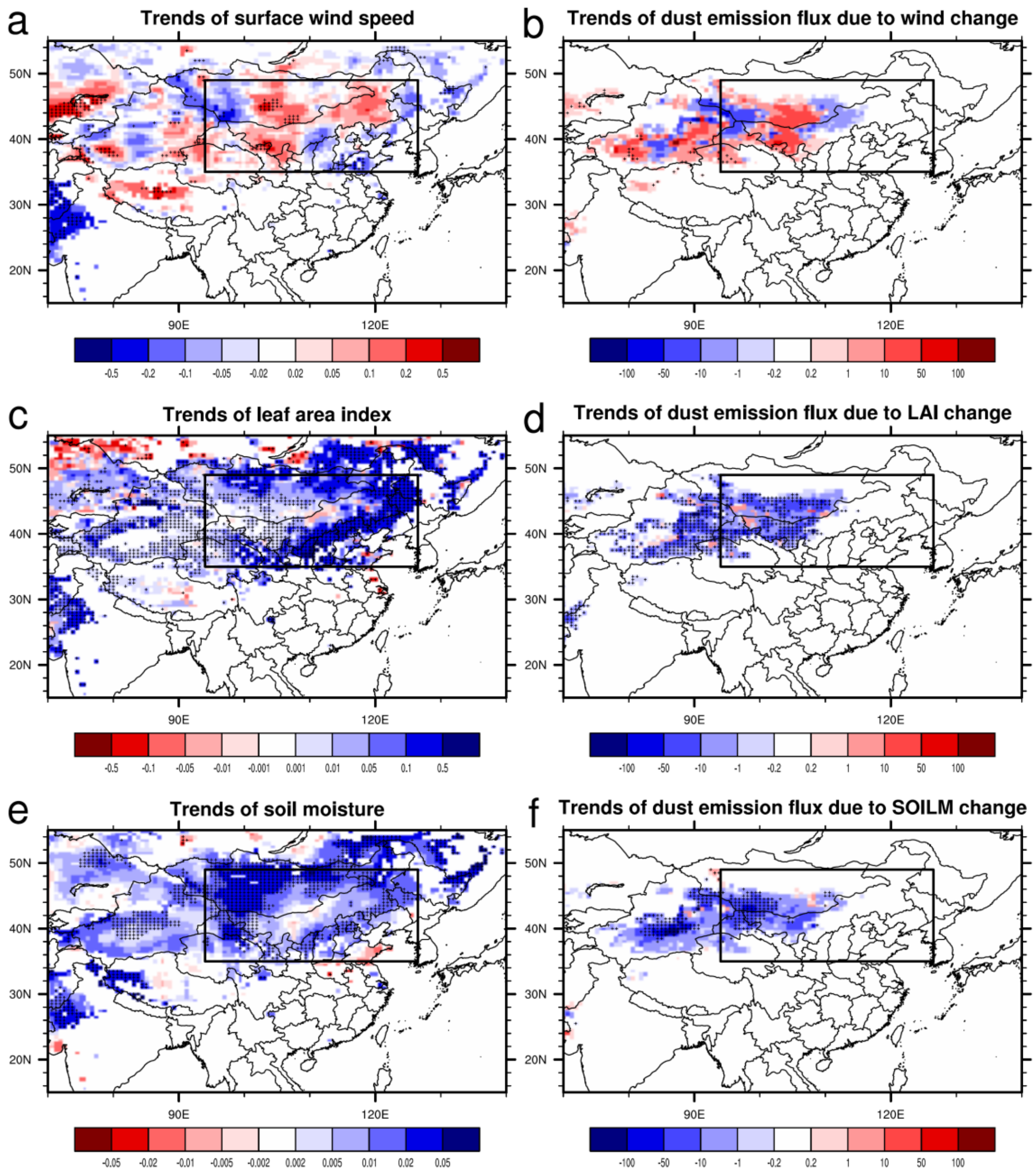

Supplementary Figure 17. Same as Figure S15, but for summer.

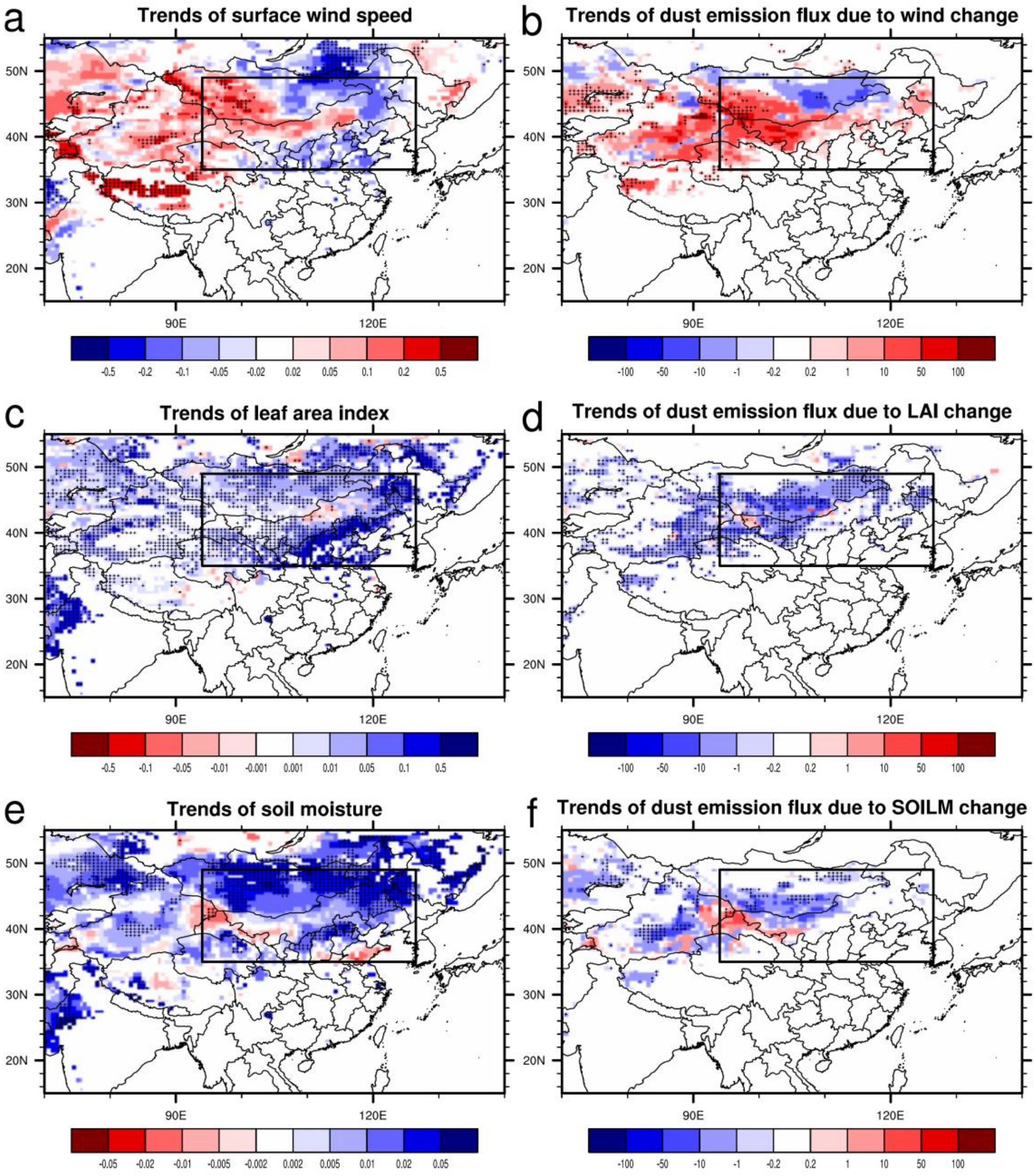

Supplementary Figure 18. Same Figure S15, but for autumn.

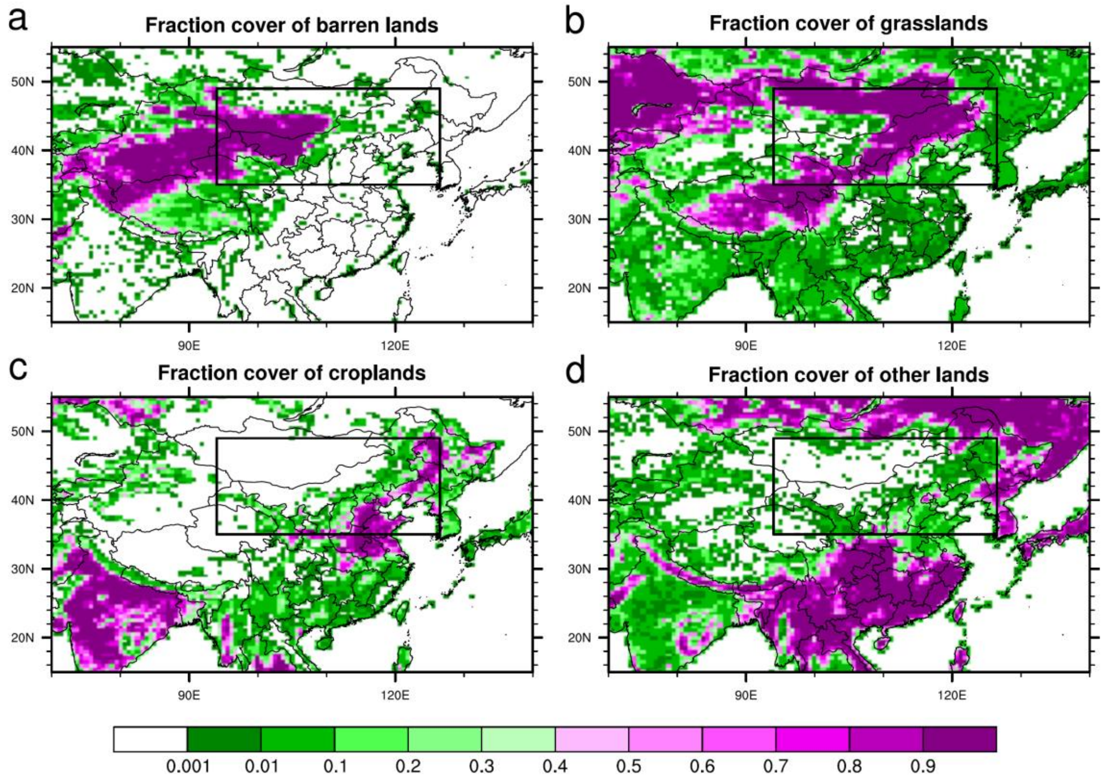

Supplementary Figure 19. Area fraction for different land cover types. (**a**) Barren lands. (**b**) grasslands. (**c**) Croplands. (**d**) Other lands. Data are from MODIS MCD12C1 dataset based on International Geosphere-Biosphere Programme (IGBP) classification (Table S3) for 2001-2017. MCD12C1 data is provided at a resolution of 0.05^{o} and aggregated into the resolution of $0.5^{o}\times 0.625^{o}$ for display here. The black rectangle denotes the Eastern Sources (35-49 ºN, 94-126.5 ºE).

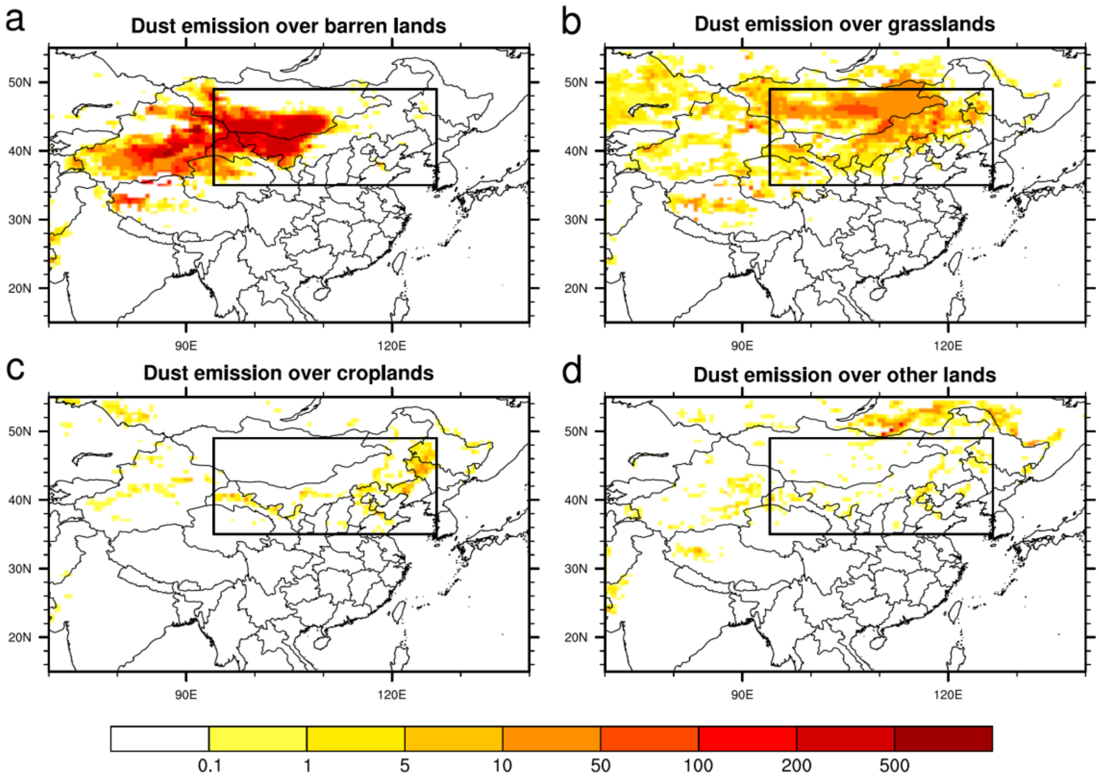

Supplementary Figure 20. Dust emission flux (g m^{-2} yr^{-1}) over different land cover types during 2001-2017. (**a**) Barren lands. (**b**) Grasslands. (c) Croplands. (**d**) Other lands. The black rectangle denotes the Eastern Sources (35-49 ºN, 94-126.5 ºE).

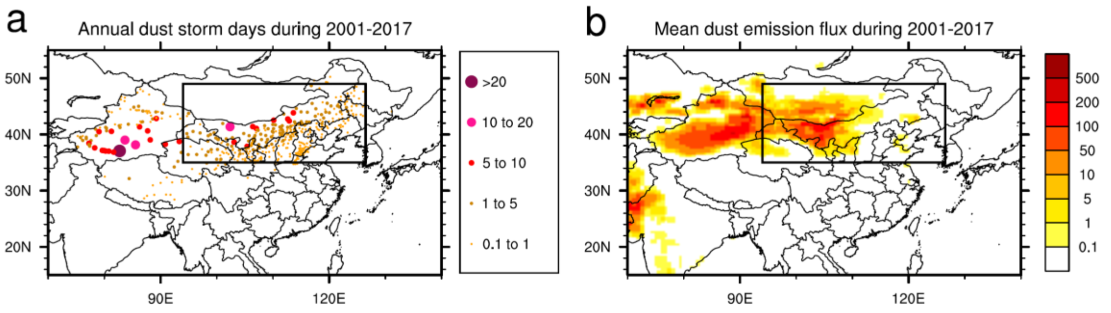

Supplementary Figure 21. Spatial distribution of observed dust storm days and simulated dust emission flux. (**a**) Annual dust storm days (N_{DS}) averaged over 2001-2017. The dust storm days are recorded at 2430 synoptic weather observations. The stations with $N_{DS} < 0.1$ days are not shown. (**b**) Annual mean dust emission flux (F_{emis}; g m^{-2} yr^{-1}) during 2001-2017 from MERRA-2 dataset. MERRA-2 reproduces the dust emission in Tarim Basin and Gobi Deserts, but it fails to capture the large dust emission regions in central-eastern part of Inner Mongolia, western part of Northeast China, and northern part of North China. The discrepancy is ascribed to the fact that MERRA-2 official dataset adopts the dust emission scheme of Ginoux et al.[35], which only calculates the dust emission over deserts. The black rectangle denotes the Eastern Sources (35-49 ºN, 94-126.5 ºE).

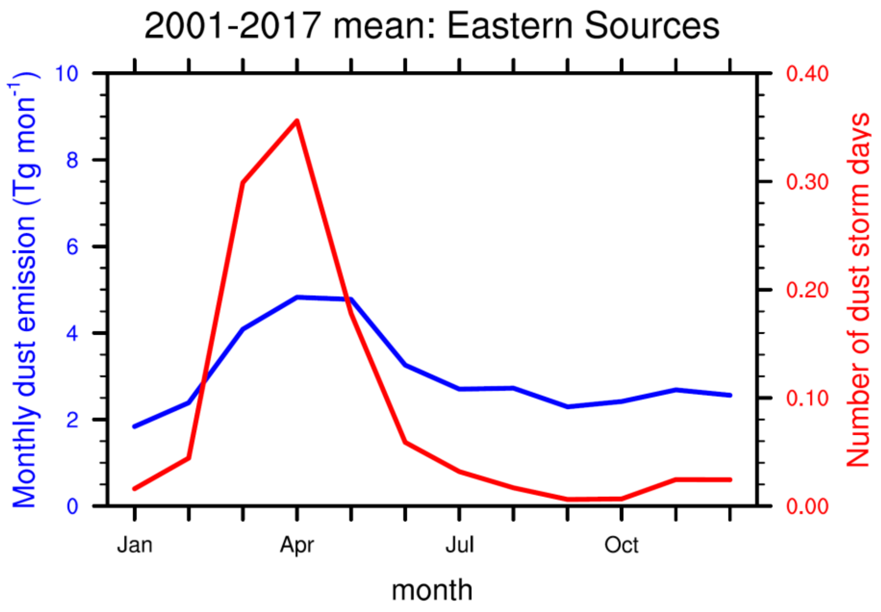

Supplementary Figure 22. Seasonal variations of dust storm days (red) and regional accumulated dust emission from MERRA-2 dataset (blue; Tg mon^{-1}) averaged over 2001-2017 for Eastern Sources. The scheme of Ginoux et al.[35] in MERRA-2 official data uses a static (i.e., invariant) soil erodibility index for potential dust sources and unable to represent the impact of dynamic vegetation on dust emission, leading to weaker seasonal variations of dust emission flux in the MERRA-2 official data.

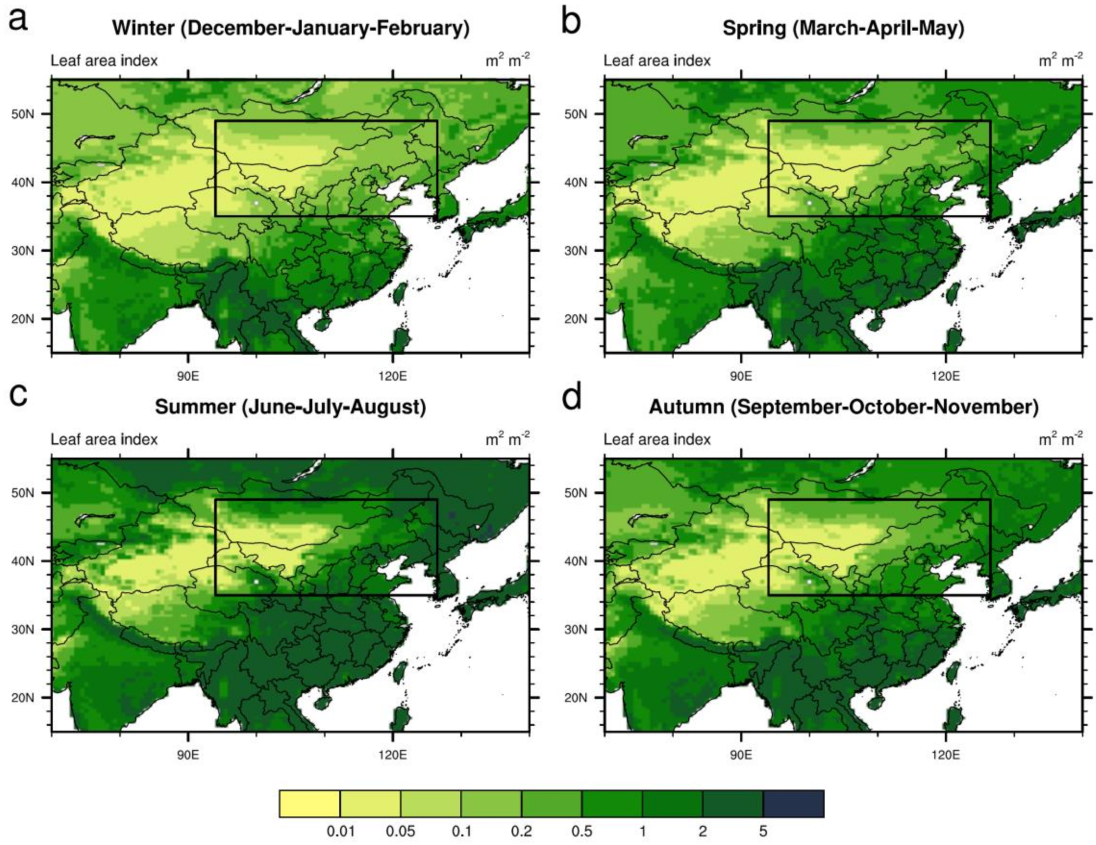

Supplementary Figure 23. Seasonal mean leaf area index (m^2 m^{-2}) in each season during 2001-2017. The black rectangle denotes the Eastern Sources (35-49 ºN, 94-126.5 ºE).

Supplementary References:

1. Shao, Y. Simplification of a dust emission scheme and comparison with data. *J. Geophys. Res.-Atmos.* **109**, D10202 (2004).
2. Shao, Y., Ishizuka, M., Mikami, M. & Leys, J. F. Parameterization of size-resolved dust emission and validation with measurements. *J. Geophys. Res.-Atmos.* **116**, D08203 (2011).
3. Shao, Y., Leys, J. F., McTainsh, G. H. & Tews, K. Numerical simulation of the October 2002 dust event in Australia. *J. Geophys. Res.-Atmos.* **112**, D08207 (2007).
4. Shao, Y., Fink, A. H. & Klose, M. Numerical simulation of a continental-scale Saharan dust event. *J. Geophys. Res.-Atmos.* **115**, D13205 (2010).
5. Kang, J. Y., Yoon, S. C., Shao, Y. & Kim, S. W. Comparison of vertical dust flux by implementing three dust emission schemes in WRF/Chem. *J. Geophys. Res.-Atmos.* **116**, D09202 (2011).
6. Hamidi, M., Kavianpour, M. R. & Shao, Y. Numerical simulation of dust events in the Middle East. *Aeolian Res.* **13**, 59-70 (2014).
7. Klose, M. et al. Mineral dust cycle in the Multiscale Online Nonhydrostatic AtmospheRe CHemistry model (MONARCH) Version 2.0. *Geosci. Model Dev.* **14**, 6403-6444 (2021).
8. Wu, C. et al. Description of dust emission parameterization in CAS-ESM2 and its simulation of global dust cycle and East Asian dust events. *Journal of Advances in Modeling Earth Systems* **13**, e2020MS002456 (2021).
9. Shao, Y. *Physics and Modelling of Wind Erosion* (Springer, Berlin, 2008).
10. White, B. R. Soil transport by winds on Mars. *J. Geophys. Res.* **84**, 4643-4651 (1979).
11. Darmenova, K., Sokolik, I. N., Shao, Y. P., Marticorena, B. & Bergametti, G. Development of a physically based dust emission module within the Weather Research and Forecasting (WRF) model: Assessment of dust emission parameterizations and input parameters for source regions in Central and East Asia. *J. Geophys. Res.-Atmos.* **114**, D14201 (2009).

12. Shao, Y. & Lu, H. A simple expression for wind erosion threshold friction velocity. *J. Geophys. Res.-Atmos.* **105**, 22437-22443 (2000).
13. Raupach, M. R., Gillette, D. A., Leys, J. F. The effect of roughness elements on wind erosion threshold. *J. Geophys. Res.-Atmos.* **98**, 3023-3029 (1993).
14. Fecan, F., Marticorena, B. & Bergametti, G. Parametrization of the increase of the aeolian erosion threshold wind friction velocity due to soil moisture for arid and semi-arid areas. *Ann. Geophys.-Atm. Hydr.* **17**, 149-157 (1999).
15. Mahowald, N. M. et al. Observed 20th century desert dust variability: impact on climate and biogeochemistry. *Atmos. Chem. Phys.* **10**, 10875-10893 (2010).
16. Oleson, K. W. et al. *NCAR Tech. Note NCAR/TN-478+STR: Technical Description of Version 4.0 of the Community Land Model (CLM)* (Natl. Cent. for Atmos. Res., Boulder, 2010).
17. Gelaro, R. et al. The Modern-Era Retrospective Analysis for Research and Applications, Version 2 (MERRA-2). *J. Clim.* **30**, 5419-5454 (2017).
18. Darmenov, A. S. Developing and testing a coupled regional modeling system for establishing an integrated modeling and observational framework for dust aerosol. (Doctoral dissertation) (Georgia Tech Library, Atlanta, 2009).
19. Reichle, R. H. et al. Assessment of MERRA-2 land surface hydrology estimates. *J. Clim.* **30**, 2937-2960 (2017).
20. Guan, Q. et al. Climatological analysis of dust storms in the area surrounding the Tengger Desert during 1960–2007. *Climate Dynamics* **45**, 903-913 (2015).
21. Guan, Q., Sun, X., Yang, J., Pan, B., Zhao, S. & Wang, L. Dust storms in Northern China: long-term spatiotemporal characteristics and climate controls. *J. Clim.* **30**, 6683-6700 (2017).
22. Zhao, Y., Xin, Z. & Ding, G. Spatiotemporal variation in the occurrence of sand-dust events and its influencing factors in the Beijing-Tianjin Sand Source Region, China, 1982–2013. *Regional Environmental Change* **18**, 2433-2444 (2018).
23. Kang, J. Y., Tanaka, T. Y. & Mikami, M. Effect of dead leaves on early spring dust emission in East Asia. *Atmos. Environ.* **86**, 35-46 (2014).

24. WMO. *WMO-No. 306: Manual on Codes* (WMO, Geneva, 2017).

25. Tai, A. P. K., Ma, P. H. L., Chan, Y.-C., Chow, M.-K., Ridley, D. A. & Kok, J. F. Impacts of climate and land cover variability and trends on springtime East Asian dust emission over 1982–2010: A modeling study. *Atmos. Environ.* **254**, 118348 (2021).

26. Kok, J. F. et al. An improved dust emission model – Part 1: Model description and comparison against measurements. *Atmos. Chem. Phys.* **14**, 13023-13041 (2014).

27. Rienecker, M. M. et al. MERRA: NASA's Modern-Era Retrospective Analysis for Research and Applications. *J. Clim.* **24**, 3624-3648 (2011).

28. Xiao, Z. et al. Use of General Regression Neural Networks for generating the GLASS Leaf Area Index product from time-series MODIS surface reflectance. *IEEE Transactions on Geoscience and Remote Sensing* **52**, 209-223 (2014).

29. Tang, H., Yu, K., Hagolle, O., Jiang, K., Geng, X. & Zhao, Y. A cloud detection method based on a time series of MODIS surface reflectance images. *International Journal of Digital Earth* **6**, 157-171 (2013).

30. Liu, Y., Liu, R. & Chen, J. M. Retrospective retrieval of long-term consistent global leaf area index (1981–2011) from combined AVHRR and MODIS data. *J. Geophys. Res.* **117**, G04003 (2012).

31. Chen, C. et al. China and India lead in greening of the world through land-use management. *Nature Sustainability* **2**, 122-129 (2019).

32. Song, Q., Zhang, Z., Yu, H., Ginoux, P. & Shen, J. Global dust optical depth climatology derived from CALIOP and MODIS aerosol retrievals on decadal timescales: regional and interannual variability. *Atmos. Chem. Phys.* **21**, 13369-13395 (2021).

33. Yu, H. et al. Interannual variability and trends of combustion aerosol and dust in major continental outflows revealed by MODIS retrievals and CAM5 simulations during 2003–2017. *Atmos. Chem. Phys.* **20**, 139-161 (2020).

34. Reichle, R. H., Liu, Q., Koster, R. D., Draper, C. S., Mahanama, S. P. P. & Partyka, G. S. Land surface precipitation in MERRA-2. *J. Clim.* **30**, 1643-1664 (2017).

35. Ginoux, P. et al. Sources and distributions of dust aerosols simulated with the GOCART model. *J. Geophys. Res.-Atmos.* **106**, 20255-20273 (2001).
